# Distribution and Functionality of Copy Number Variation across European Cattle Populations

**DOI:** 10.3389/fgene.2017.00108

**Published:** 2017-08-23

**Authors:** Maulik Upadhyay, Vinicus H. da Silva, Hendrik-Jan Megens, Marleen H. P. W. Visker, Paolo Ajmone-Marsan, Valentin A. Bâlteanu, Susana Dunner, Jose F. Garcia, Catarina Ginja, Juha Kantanen, Martien A. M. Groenen, Richard P. M. A. Crooijmans

**Affiliations:** ^1^Animal Breeding and Genomics, Wageningen University and Research Wageningen, Netherlands; ^2^Department of Animal Breeding and Genetics, Swedish University of Agricultural Sciences Uppsala, Sweden; ^3^Institute of Zootechnics and Nutrigenomics and Proteomics Research Center, Università Cattolica del Sacro Cuore Piacenza, Italy; ^4^Institute of Life Sciences, Faculty of Animal Science and Biotechnologies, University of Agricultural Sciences and Veterinary Medicine of Cluj-Napoca Cluj-Napoca, Romania; ^5^Department of Animal Production, Veterinary Faculty, Universidad Complutense de Madrid Madrid, Spain; ^6^Departamento de Apoio, Produção e Saúde Animal, Faculdade de Medicina Veterinária de Araçatuba, Universidade Estadual Paulista Araçatuba, Brazil; ^7^IAEA Collaborating Centre on Animal Genomics and Bioinformatics Araçatuba, Brazil; ^8^Centro de Investigação em Biodiversidade e Recursos Genéticos (CIBIO-InBIO), Universidade do Porto Vairao, Portugal; ^9^Green Technology, Natural Resources Institute Finland Jokioinen, Finland; ^10^Department of Environmental and Biological Sciences, University of Eastern Finland Kuopio, Finland

**Keywords:** copy number variations, European cattle, high density SNP array, population differentiation, purifying selection, drift, *Kit* gene

## Abstract

Copy number variation (CNV), which is characterized by large-scale losses or gains of DNA fragments, contributes significantly to genetic and phenotypic variation. Assessing CNV across different European cattle populations might reveal genetic changes responsible for phenotypic differences, which have accumulated throughout the domestication history of cattle as consequences of evolutionary forces that act upon them. To explore pattern of CNVs across European cattle, we genotyped 149 individuals, that represent different European regions, using the Illumina Bovine HD Genotyping array. A total of 9,944 autosomal CNVs were identified in 149 samples using a Hidden Markov Model (HMM) as employed in PennCNV. Animals originating from several breeds of British Isles, and Balkan and Italian regions, on average, displayed higher abundance of CNV counts than Dutch or Alpine animals. A total of 923 CNV regions (CNVRs) were identified by aggregating CNVs overlapping in at least two animals. The hierarchical clustering of CNVRs indicated low differentiation and sharing of high-frequency CNVRs between European cattle populations. Various CNVRs identified in the present study overlapped with olfactory receptor genes and genes related to immune system. In addition, we also detected a CNV overlapping the *Kit* gene in English longhorn cattle which has previously been associated with color-sidedness. To conclude, we provide a comprehensive overview of CNV distribution in genome of European cattle. Our results indicate an important role of purifying selection and genomic drift in shaping CNV diversity that exists between different European cattle populations.

## Introduction

Copy number variation (CNV), which is defined as large-scale losses and gains of DNA fragments, forms one of the major classes of genetic variation (Zhang et al., 2009). In terms of total bases involved, CNV affects a larger fraction of genome compared to Single Nucleotide Polymorphisms (SNP) (Redon et al., 2006). In addition, estimates from several autosomal dominant diseases have indicated a higher *de novo* locus-specific mutation rate for CNV than for SNP (Lupski, 2007a). Copy number losses and gains of genetic sequences roughly make up 5–10% of the human genome, and many of the regions affected are associated with phenotypic variations including susceptibility to specific diseases (Redon et al., 2006; Stankiewicz and Lupski, 2010; Zarrei et al., 2015). For example, the presence of lower *CCL3L1* gene copy number compared to the population average is associated with contracting HIV and developing AIDS (Gonzalez et al., 2005). Differences in copy number of genomic segments can result in changes in gene expression and phenotypic variation through gene disruption and altering gene dosage. For example, duplication of the *APQ7* gene in human has been linked with the emergence of traits related to endurance running as a consequence of increased expression (Dumas et al., 2007; Lupski, 2007b).

A number of studies have been undertaken in various domestic animals to characterize CNV and their effects on phenotypes (Chen et al., 2012; Liu et al., 2013; Bickhart et al., 2016; Zhu et al., 2016). For example, duplication of a set of fibroblast growth factor (*FGF*) genes and the *ORAOV1* gene in Rhodesian and Thai Ridgeback dogs causes a characteristic dorsal hair ridge (Salmon Hillbertz et al., 2007). A partial or complete duplication of the *KIT* gene causes different patterns of white coat coloration in pigs and in some of the cattle breeds such as White park, Galloway and Belgian blue (Pielberg et al., 2002; Durkin et al., 2012; Brenig et al., 2013). Similarly, white coat color in sheep has been associated with a duplication of the *ASIP* gene (Norris and Whan, 2008). Because CNV is known to be common in regions of the genome that regulate important physiological functions, they have also been studied for association with economically important traits in domesticated animals, such as milk production and fertility. For example, several CNVs associated with milk production traits in Holstein Friesian (HF) cattle have been identified (Xu et al., 2014).

The aurochs (*Bos primigenius primigenius)* is the ancestor of European cattle. Although the wild ancestor no longer exists, many extant European cattle breeds still possess primitive, aurochs-like features. These breeds are often referred to as primitive cattle breeds (Upadhyay et al., 2016). By contrast, commercial cattle breeds, including Holstein-Friesian (HF), Brown Swiss (BS) and Jersey, display derived phenotypic traits such as polledness and early maturity. It is likely that some of these differences in traits between primitive and modern cattle may result from CNV. Systematically assessing CNV between commercial and primitive cattle breeds might, therefore, reveal the genetic changes responsible for phenotypic differences, which have accumulated throughout the domestication history of cattle as result of natural and artificial selection. Moreover, contrasting cattle populations from different European regions may provide insight into the role of CNVs in population differentiation. Also, as previous studies (Park et al., 2015; Upadhyay et al., 2016) have indicated a higher frequency of aurochs specific alleles in North-western European cattle breeds compared to Balkan and Italian cattle breeds probably as a result of secondary contact between local aurochs and ancestor of domestic cattle, it is possible that comparing CNV patterns between cattle of different regions may reveal unique CNV that might have been introgressed into the ancestors of present domestic European cattle during this secondary contact. Hence, the objectives of the current study are to provide comprehensive overview of CNV distribution in various European cattle populations and to carry out comparative evaluation of CNV patterns between cattle populations originating from different regions of Europe and thus, to address the role of CNVs in population differentiation.

## Materials and Methods

### Sample Collection and Genotyping

A total of 196 animals from 38 different cattle breeds, consisting mainly of primitive cattle breeds (27 breeds), were sampled. Numbers of animals per breed varied from 1 to 6 (**Supplementary Table S1**), except for Holstein-Friesian (HF), for which 55 animals were used to identify CNVs. The geographic origin of 189 animals was assigned to one of five regions or breed groups: British and Irish (BRI), Dutch (NLD, ancestral to large parts of the Lowland Pied and Baltic Red cattle), Balkan and Italian (BAI, representing Podolian and Busha cattle), Iberian (IBR), and Alpine (ALP, combining the Central Brown and Spotted breed cluster). Because five Heck (HE) cattle did not belong to any of these groups and geographic origin of two samples were not assigned confidently they were not categorized in any of these five groups.

DNA was extracted from hair roots, blood or semen. Hair and blood samples were collected by a veterinarian in accordance with EU legislation. Semen samples were obtained from commercial AI services. The research did not involve experimentation on animals requiring approval of Animal Experiments Committee (DEC), Netherlands. The samples were genotyped with the Illumina BovineHD Genotyping BeadChip, which contains 777,692 SNPs uniformly spanning the bovine genome. All the SNP were clustered and analyzed using the Illumina BEADSTUDIO software (2.0).

### Identification of CNVs

We used the PennCNV software to identify CNVs (Wang et al., 2007). A Hidden Markov Model (HMM) algorithm as employed in PennCNV incorporates multiple parameters, such as total signal intensity (LRR), allelic intensity ratio (BAF) values of each marker for each individual, and the population frequency of B allele (PFB) of SNPs. Both the LRR and BAF values of each marker for each sample were generated from the Illumina Genome Studio software package using the default clustering file (Illumina Inc, United States). The PFB was calculated as the average BAF for each marker in this population. We only used autosomal markers for detection of CNVs. The chromosomal positions of the SNPs were derived from the bovine UMD3.1 genome sequence assembly. To reduce the number of false positives, the LRR of each SNP was adjusted for the GC content of 1 Mb window surrounding the SNP using the ‘-gcmodel’ option as employed in PennCNV. The PennCNV algorithm (with options: -test) was applied to all 29 autosomes (with option: -lastchr 29) to detect cattle CNV. All samples with standard deviation of LRR > 0.30, standard deviation of BAF > 0.001 and wave factor > 0.05 were discarded for downstream analysis. Finally, 47 low quality samples were discarded from further analysis. Of the remaining samples (**Supplementary Table S1**), a CNV was included in the downstream analysis if it spanned minimum of 3 SNPs (default). Furthermore, a CNV region (CNVR) was defined as a union of overlapping CNVs detected in two different samples (Redon et al., 2006). The identification of CNVRs was performed using a custom python script.

### Comparison of Cumulative CNV Counts and CNV Size

To compare differences in CNV counts and CNV size between the five major breed groups, we first removed the samples showing outlier values (mean ± 3 standard deviations) for either CNV counts, or CNV size, or both, if more than five samples represented a breed. In the second step, we used the Kruskal–Wallis test to assess overall differentiation of cumulative CNV counts, and One Way ANOVA to assess overall differentiation of cumulative CNV size among the five breed groups of cattle. If the overall *P*-value was significant (*P* < 0.05), we performed a *post hoc* Mann–Whitney test for cumulative CNV counts and a *t*-test for cumulative CNV size to assess pair-wise population differences followed by Bonferroni correction for multiple testing.

### qPCR Validation

Copy number variations were validated by Real-time qPCR using the 7500 Fast and RT (Real-time) PCR system (Applied Biosystems). Primers were designed using the Primer3 webtool^1^. Information about Primers and samples used in qPCR are given in **Supplementary Table S2**. All PCR primers were designed from UMD 3.1 reference genome based on the first 1000 bp regions of CNVs. Before PCR, the quality and quantity of every DNA sample was measured with Qubit^®^2.0 Fluorometer. PCR amplifications were performed in a total volume of 12.5 μL containing reagents described in **Supplementary Table S2**. The BTF3 gene was chosen as internal standard in all qPCR experiments.

### Hierarchical Clustering of CNVR Data

To cluster samples according to their CNV similarities, we made a vector of “0”s and “1”s for each individual based on presence or absence of a specific CNVR in that particular individual. A hierarchical clustering was performed using the *DendroUPGMA*.^2^ We used Jaccard index as a distance measure and the unweighted pair-group method with average mean (UPGMA) as the clustering method.

### Identification of Segmental Duplications

We applied Whole Genome Assembly Comparison (WGAC) to identify intrachromosomal segmental duplications as described previously (Khaja et al., 2006). In the first step, we retrieved the Repeat-masked UMD 3.1 cattle genome assembly from the UCSC database^3^. Subsequently, we used MegaBlast to perform sequence similarity searches within the assembly. Finally, we retrieved all paralogous sequences which displayed >90% similarity and with a size of >5 Kb.

### Assessment of Population Differentiation Using V_st_

To detect population differentiated CNVs between cattle populations of different regions, V_st_ was calculated. We calculated pair-wise V_st_ as defined previously (Redon et al., 2006) by using the equation: (V_s_-V_T_)/ V_T_, where V_T_ is the total variance in mean of LRR of a probe across all individuals of two populations and V_s_ is the average variance of a probe in samples within each breed. We used a window-based approach to identify groups of minimally 3 SNP probes, each showing significant V_st_ (V_st_ > 0.35) with the window shift of a single SNP probe. Finally, we only referred to a CNV as population differentiated if it contained the group of SNPs identified as having significant V_st_ in this window based approach.

### Gene Contents and Functional Annotation

The unique cattle gene list based on UMD 3.1 was retrieved from Ensembl biomart (Cow release 84). The PANTHER classification system was used to assess the probability of overrepresented genes in CNVRs within biological process, cellular component, and molecular function using Bonferroni correction for multiple comparisons (Mi et al., 2005).

## Results

### Overview of Copy Number Variation (CNV) across All Groups

**FIGURE 1:**
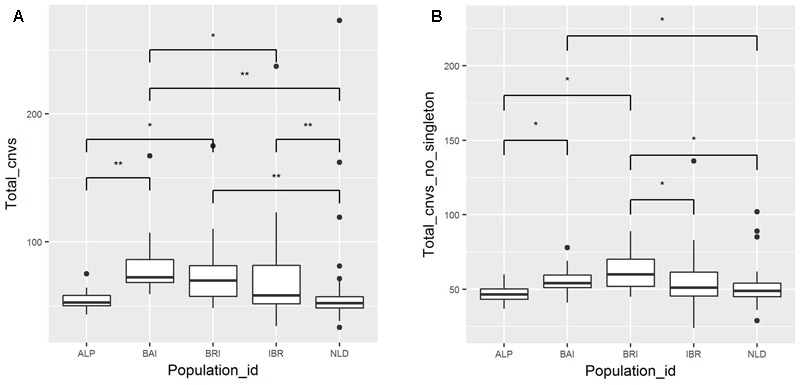
Number of detected CNVs per sample. Samples are categorized based on their origins. The median of the population is indicated by central line in a box, while the black dots represents outliers. Connecting bar above box plots between pair of populations displays significant *P*-values. “^∗^” Indicates *P* < 0.05 and “^∗∗^” indicates *P* < 0.01. **(A)** Total CNV counts with singleton. **(B)** Total CNV abundance excluding singleton.

A total of 9,944 autosomal CNVs were identified in 149 European cattle samples (**Supplementary Table S3**). Out of 9,944 CNVs, 1,941 were identified as singletons (**Supplementary Table S4**), while the remaining CNVs had minimally a base overlap with at least one CNV identified in another sample. The average number of CNV identified per sample was 67. For the different breed groups, BAI, BRI, IBR, NLD, ALP, the average number of CNV per sample was 80, 79, 70, 58, and 55 respectively. We found overall significant differences (*P* < 0.05, Kruskal–Wallis test) as well as pair-wise population differences (*P* < 0.05, *post hoc* Mann–Whitney test) in the average number of identified CNV per individual in each of the five major breed groups (**Figure 1A**). Excluding singletons from the comparison also resulted in significant differences in average number of CNVs between the major breed groups (**Figure 1B**). On the other hand, despite overall significant differences (*P* < 0.05, One way ANOVA) in average cumulative length of CNVs per individual in each of the five major breed groups, pair-wise *post hoc t*-test did not result in a significant difference between any of these groups.

### CNV Validation Using qPCR

To validate CNVs identified in the present study, we performed quantitative PCR (qPCR) assays for 18 CNV loci in 33 animals. These loci were chosen to represent different copy number states (loss, gain and complex) and different frequency ranges (from singleton to multiple individuals). Of the 18 CNVs tested, 13 could be confirmed by qPCR. The copy number estimated by qPCR for all confirmed CNVs, agreed with the state estimated by the PennCNV analysis. Of the 5 non-validated CNVs, 3 were identified only in one animal, while the remaining 2 were identified in at least two animals. Only 1 of the 5 non-validated CNVs did not amplify because of the poor DNA quality of samples, while non-amplification of the remaining CNVs indicated normal copy instead of hemizygous deletion as identified by PennCNV for all locus. These results also indicate high likelihood of singleton CNVs, i.e., CNVs that occurs only once in the dataset, being false positive (**Supplementary Table S2**).

### Overview of CNVRs

**FIGURE 2:**
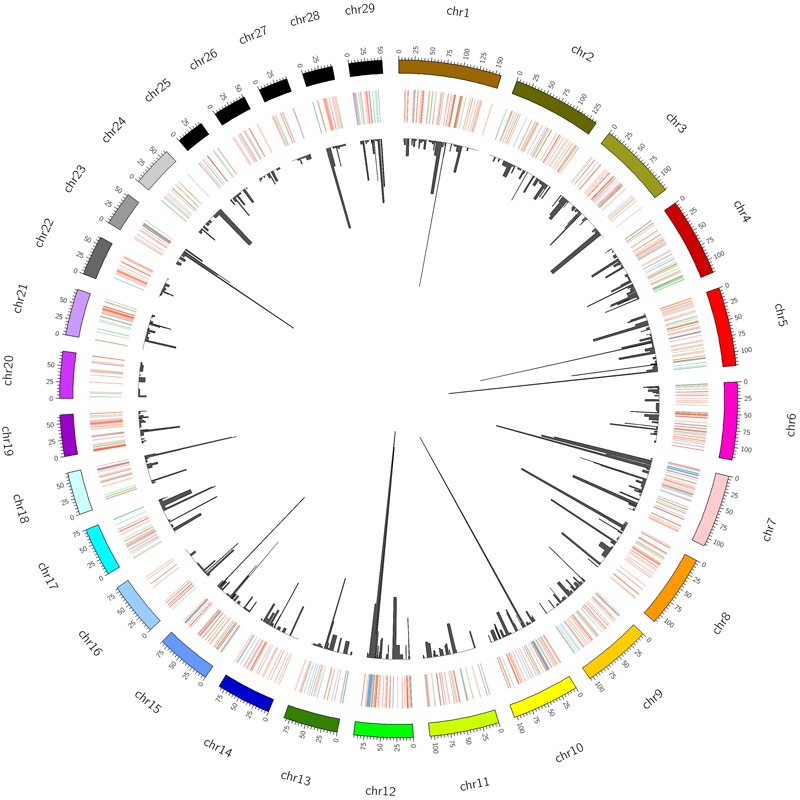
Distribution, status and frequency of CNVR in the bovine genome (UMD 3.1). The status of CNVRs are shown in outer circle in Red (loss), Green (gain) and Blue (both), while the inner circle is indicative of the frequency.

**FIGURE 3:**
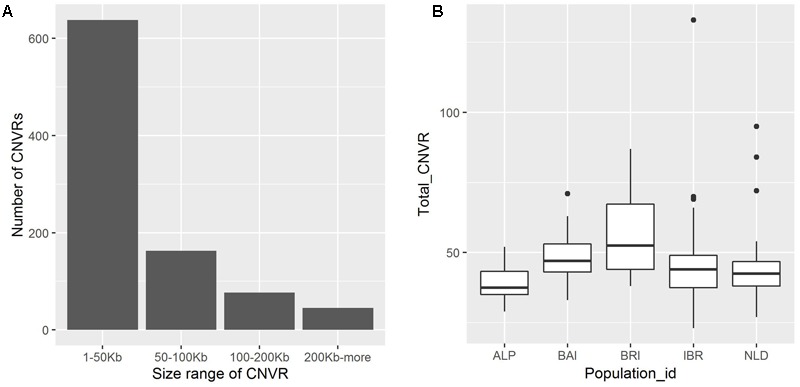
**(A)** Distribution of CNVR size in the genome. **(B)** Distribution of CNVR per sample categorized based on its origin. The median of the population is indicated by central line in a box, while the black dots represents outliers.

A total of 923 CNVRs were identified by aggregating overlapping CNVs with overlap identified in at least two animals (**Figure 2**, **Supplementary Table S5**). These CNVRs cover 61.06 Mb of the cattle UMD 3.1 genome assembly, which corresponds to ∼2.5% of the 29 bovine autosomes. However, the distribution of CNVRs across all autosomes varies considerably, with the highest number (55) on chromosome 1 and the lowest (10) on chromosome 27. The estimated length of CNVRs varies from 1.53 Kb to 3.51 Mb, with an average of 66.15 Kb (**Figure 3A**). The ratio of total estimated CNVR length per chromosome to the length of that chromosome varies from 8.20% for chromosome 12–0.040% for chromosome 26. Chromosome 23 displays the highest density of CNVRs with an average distance of 1.46 Mb between CNVRs, while chromosome 20 exhibits the lowest density of CNVRs with an average distance of 5.24 Mb between CNVRs. These 923 CNVRs are comprised of 587 losses, 179 gains and 157 complex (both loss and gain) events. Furthermore, the frequency of these CNVRs in the populations under study ranged from 1.34% (present in 2 of the 149 animals) to 97.31% (present in 145 of the 149 animals). The 157 complex CNVRs, on average, displays much higher frequencies (∼13%) than the average frequency of only losses (∼3.6%) or only gains (∼3.4%) CNVR events. One complex CNVR (id: CNVr527) displays the highest frequency (97.31%) and is located on chromosome 12 between 73.2 and 76.7 Mbp. Out of all 923 CNVRs, 198 CNVRs (21%) have a frequency of more than 5% in the studied cattle populations (**Supplementary Table S5**). Of these 198 CNVRs, 49 were identified in at least one individual of each of the five breed groups (**Supplementary Table S5**). The BRI and NLD populations revealed the highest (26) and the lowest (8) number of CNVRs per sample respectively (**Figure 3B**).

### Hierarchical Clustering Based on CNVRs

To assess population differentiation, hierarchical clustering was carried out after converting CNVRs into binary data (see Materials and Methods). Samples belonging to low diversity breeds, as identified in our previous SNP based studies (Upadhyay et al., 2016), such as English longhorn (EL) and Boskarin (BK), cluster together (Supplementary Figure S1). However, samples belonging to IBR breeds show a lower degree of differentiation compared to samples belonging to the other regions. Also, the breeds from the same geographic region do not cluster together. For example, BS (an Alpine breed) samples form a clade with the Iberian samples belonging to Pajuna (PA), indicating sharing of high frequency CNVRs.

### Association between High Frequency CNVRs and Intra-Chromosomal Segmental Duplications (SD)

The large proportion (>21% of total) of CNVRs displaying a frequency above 5% indicates that either; (1) they fall into regions of CNV hot spots in the cattle genome, (2) these are likely to be of more ancient origin when compared to the low frequency CNVRs or (3) they are under strong positive selection. CNV hot spot regions in the genome usually overlap with genomic segmental duplications (SDs) (Sharp et al., 2005). Thus, to assess the correlation between SDs and high frequent CNVRs, we identified SDs from the UMD 3.1 bovine genome assembly. In the analysis, we only considered intra-chromosomal SDs longer than 5 Kb, as these SDs are more likely to cause misalignment and aberrant recombination (Stankiewicz and Lupski, 2002) than the small size SDs (<5 Kb). Of the 21 CNVRs that overlapped with large SDs, 14 were present at a frequency of more than 5% across all cattle populations (**Supplementary Table S6**).

### Assessment of Population Differentiated CNVs Based on V_st_

To identify CNVs contributing to population differentiation, we calculated the pairwise V_st_ between every possible combination of the five major breed groups, and between, HF and the four major breed groups. In the latter V_st_ analysis comparing HF samples with other breed groups we did not include NLD, as large proportion of its sample size consisted of HF samples. The value of V_st_ varies between 0 (no population differentiation) and 1 (complete population differentiation), with high V_st_ values suggesting a difference between populations in the frequencies of copy number states of underlying sequences. Interestingly, we observed a very few breed group differentiated CNVs for all combinations except for HF vs. BRI, where we observed quite a few breed group differentiated CNVs. We also considered the effect of mis-assembly on lineage differentiated CNVs as it has been reported previously that incorrect placement of the sequence from the sex chromosome on autosomes may distort the dosage ratio between male and female which can lead to false positive lineage population differentiated CNVs (Zhou et al., 2016). Clearly, except for three CNVRs, all CNVRs identified as breed group differentiated (in HF vs BRI) displayed difference in LRR values between bull and cow samples (Supplementary Figure S2 and **Table S7**). Thus, the high V_st_ observed between BRI and HF for CNVs involving miss-assembled SNPs can be attributed to the highest proportion of female samples in the BRI group, while all HF samples were male.

### Gene Content of CNVRs

Of the 923 CNVRs identified in the present study, 415 (∼45%) span (with at least one bp overlap) 916 unique cattle genes (**Supplementary Table S8**). Interestingly, genes from certain immune response related gene families such as the *TRAV* like gene family, and *IGL* were covered by complex CNVRs indicating differences in copy number between different cattle breeds. The most frequent CNVR (id:CNVr527) covered four related genes, which are located in tandem and display similarity to *ABCC4* in human. We also found genes related to economically important traits of livestock covered by CNVRs such as *MTHFSD*, and *GTF2I*. In addition, CNVRs also covered some essential genes such as *MSH4* and *ATF2* (**Supplementary Table S9**). The gene ontology (GO) analysis for 916 ensemble unique genes using the Panther classification system (REF) showed that terms related to immunity and olfactory activity were overrepresented in the CNVRs that were identified in the present study (**Supplementary Table S10**).

**FIGURE 4:**
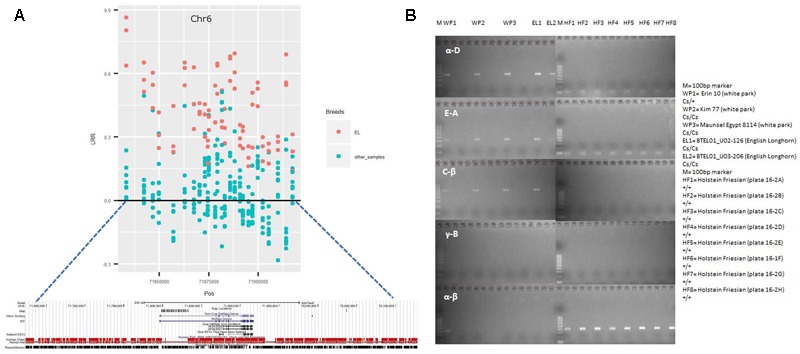
CNVr2375 completely overlaps the *Kit* gene in English longhorn (EL) samples. **(A)** Regional SNP plot of CNVr2375. The mean LRR for each marker in EL samples are displayed in Red, while that of the remaining samples are displayed in light green. **(B)** Result of PCR performed to validate the presence of Cs29 allele in EL and White Park cattle, where α-D, E-A, and C-β refers to the fusion points of Cs29 allele (for more details refer to Durkin et al., 2012), while α- β refers to the wild types (+) allele. WP, White Park cattle; EL, English Longhorn cattle; M, 100 bp ladder Marker; HF, Holstein-Friesian.

Interestingly, 5 cattle samples displayed a CNVR (id: CNVr2375) covering the *KIT* gene. However, occasionally, CNVs contributing to a CNVR may or may not fall in the gene covered by that CNVR. Hence, we investigated the CNVs within CNVr 2375. Detailed analysis of these CNVs revealed that of the 5 samples, 3 EL samples had a CNV gain covering the *KIT* gene, while the remaining samples had CNVs outside the gene. In addition, the estimated size and position of this CNV within CNVr 2375 (**Figure 4A**) displayed similarity with the genomic segment involved in a serial translocation from chromosome 6 to chromosome 29 that was previously reported in Belgian Blue, Galloway and White Park cattle breeds (Durkin et al., 2012; Brenig et al., 2013). Thus, to test the presence of that same serial translocation overlapping the *KIT* gene, we PCR amplified the known fusion points of the translocation (for more details refer to Durkin et al., 2012). The amplification of fusion points (α-D,E-A and C-β) confirm the presence of the Belgian Blue type allele (Cs29) in 2 EL samples (**Figure 4B**), whereas the remaining sample did not amplify due to poor DNA quality. The White Park cattle samples, which were discarded from the analysis due to high standard deviation in LRR, also reveal the presence of the Cs29 allele. These results show a high prevalence of the Cs29 allele in BRI cattle breeds.

## Discussion

### Difference in Abundance of CNV Counts between Different Cattle Populations

**FIGURE 5:**
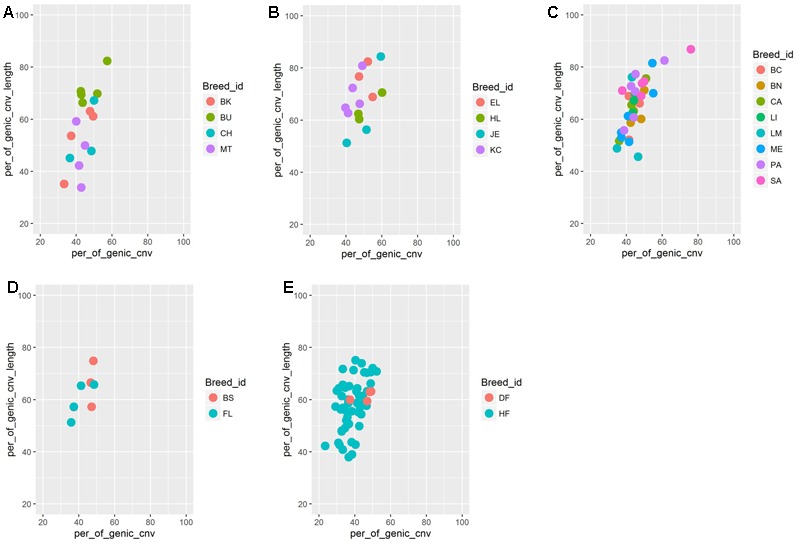
Percentage of genic CNV counts (on *X* axis) and cumulative genic CNV length (on *Y*-axis) per individual split based on breed and its geographical region. **(A)** Balkan and Italian cattle breeds (BAI), **(B)** British and Irish cattle breeds (BAI), **(C)** Iberian cattle breeds (IBR), **(D)** Alpine cattle breeds (ALP), and **(E)** Dutch (NLD) cattle breeds

We found significant differences in CNV counts between different cattle populations. The BAI and the BRI breed groups displayed relatively high number of CNVs per individual compared to the breed groups from other regions. The mean CNV cumulative length per individual between different cattle groups, however, was not significantly different. Such difference in CNV abundance between different cattle populations have already been reported previously. For instance, high CNV abundance has been reported in indicine and African taurine cattle breeds than in European taurine, which has been attributed to their breed divergence and population history (Liu et al., 2011). Similar observations were also reported in other species as well. For instance, Pezer et al. (2015) reported difference in total CNV abundance and total genic deletions between several natural populations of the house mouse, which they attributed to difference in effective population size between mouse populations. It is evident from these studies that population history such as change in past effective population size, gene flow, and selection process may contribute to differential CNV abundance between different populations. Thus, we hypothesize that persistence of small effective population size over many generations in BRI breeds such as EL and Highland (HL) may have resulted in relaxation on purifying selection against slightly deleterious CNVs and which in turn, may have resulted in accumulation of large number of CNVs and genic deletion events. To test this hypothesis, we calculated the number of genic deletion CNVs, percentage of genic CNVs as well as cumulative genic CNV length in breeds with more than or equal to 3 samples. (**Figure 5** and Supplementary Figure S3). Indeed, we observed a higher number of genic deletion as well as higher cumulative length of genome under deletion in BRI breeds [English Longhorn (EL) and Highland (HL) (Highland)] compared to breeds from other regions. This observation supports the hypothesis that genetically isolated small populations may accumulate abundance of CNVs, in particular, deletion CNVs. However, we note that, since some SNP arrays display bias toward detection of deletions (Pinto et al., 2011) and the present study suffers from low sample size per breed, large samples from multiple breeds are needed to be sampled to explore this hypothesis further.

On the other hand, within population variability in number of genic and total number of CNVs in several BAI and IBR breeds may be attributed to high admixture pattern of their genomes (Decker et al., 2014; Upadhyay et al., 2016) or higher historical effective population size compared to NLD, ALP or BRI breed-groups.

### Comparison of Identified CNVRs with Previous Studies

To characterize the CNVRs identified in the present study in more detail, we compared them to the CNVRs identified in eighteen previous studies using various methods such as whole genome sequencing (Stothard et al., 2011; Boussaha et al., 2015; Bickhart et al., 2016; Ben Sassi et al., 2016), comparative genomic hybridization (Fadista et al., 2010; Liu et al., 2010; Kijas et al., 2011; Zhang L. et al., 2014; Zhang et al., 2015), Illumina Bovine HD BeadChip Arrays (Hou et al., 2012; Jiang et al., 2013; Zhang Q. et al., 2014; Sasaki et al., 2016), and Illumina Bovine 50K SNP array (Bae et al., 2010; Hou et al., 2011; Jiang et al., 2012). The comparisons revealed that 737 (80% of the total) number of CNVRs detected in the present study overlapped completely or partially (by at least a single bp overlap) with CNVRs from these previous studies detected in literature (**Table 1**). The inconsistency of overlaps with different studies can be attributed to differences in size and structure of populations under investigation, platforms and algorithms of CNV calling, definitions of CNV and CNVR between various studies. As expected, high overlaps are reported in studies that have investigated CNVs in diverse cattle breeds using the Illumina Bovine HD BeadChip array (**Table 1**).

**Table 1:** Comparison between CNVRs identified in the present study with previous studies in terms of count and length.

Methods	Study	Total CNVR segment	Total cumulative CNVR length (in bp)	Overlapped CNVR	Overlapped CNVR (%)	Overlapped CNVR cumulative length (%)
50K	Bae et al., 2010	368	63,107,899	39	10.60	3.36
	Hou et al., 2011	743	15,8021,612	188	25.30	15.24
	Jiang et al., 2012	101	23781,338	19	18.81	3.63
	Wang et al., 2015	389	70748371	60	15.42	3.40
770K	Hou et al., 2012	3438	146902512	658	19.14	25.97
	Jiang et al., 2013	357	34407298	119	33.33	47.32
	Sasaki et al., 2016	861	43654930	347	40.30	48.30
	Xu et al., 2016	257	12443243	95	36.96	80.11
	Zhang Q. et al., 2014	365	13128818	116	31.78	53.60
CGH	Fadista et al., 2010	254	15760830	22	8.66	7.94
	Kijas et al., 2011	27	6085066	6	22.22	9.98
	Liu et al., 2010	200	36171861	91	45.50	41.32
	Zhang L. et al., 2014	353	42915082	41	11.61	9.14
	Zhang et al., 2015	339	36596362	35	10.32	9.23
WGS	Bickhart et al., 2012	1265	55590961	87	6.88	7.90
	Boussaha et al., 2015	4199	1012466378	1314	31.30	41.83
	Ben Sassi et al., 2016	823	45420220	89	10.81	26.47
	Stothard et al., 2011	790	3287618	48	6.08	4.93

*The term “overlap” refers to the number or percentage of CNVR of previous study that display overlap with the present study. Abbreviations: 50K-Illumina BovineSNP50 BeadChip, 770K-Illumina BovineHD BeadChip, CGH, Comparative Genomic Hybridization; WGS, Whole Genome Sequencing.*

The identification of 186 Novel CNVRs suggests that a substantial number of CNVs in cattle genomes are yet to be identified (**Supplementary Table S11**). Of the 186 CNVRs (∼20% of the total) identified as novel in this study, 49 are breed specific CNVRs for various breeds. Of the 49 private CNVRs, 27 are observed only in HF. We note that HF might have shown the highest percentage of breed specific CNVRs due to the larger sample size investigated in our study. However, despite the small sample size, all BRI breeds displayed at least one breed specific CNVR. Interestingly, the EL displayed quite a few breed specific private CNVRs (6) followed by Heck (HE), which displayed 4 breed specific CNVRs.

### Sharing of Highly Frequent CNVRs and Low Differentiation between European Cattle Populations

Hierarchical clustering of CNVRs revealed that animals which belonged to breeds with a relatively low diversity such as EL, Boskarin (BK), Dutch Friesian (DF), Maltese (MT), and Heck (HE) formed a clear cluster. This type of a clustering pattern suggests that low diversity led to an increase in shared CNVRs between animals. For example, the Boskarin (BK) breed displayed more than 40% of the total CNVRs as shared between two or more samples (data not shown). However, unlike SNP based clustering of these same samples in our previous study (Upadhyay et al., 2016), samples from the same region did not cluster together (Supplementary Figure S1). This indicates a relatively low level of differentiation or sharing of high frequent CNVRs among different European cattle breeds. This discordance in inference of European cattle population structure indicates that either our analysis suffered from low sample size per breed or most CNVs are transient enough to not have followed the same pattern of demographic events in the history of cattle domestication that typical neutral genetic variants have experienced. In addition, it can be speculated that de-novo CNVs, CNV hot-spot regions in the genome and false CNV calls due to variation in genotyping intensities can also affect inference of population stratification.

To investigate the highly frequent CNVRs in more detail, we performed their association with SDs. It has already been shown that SDs provide substrate for non-allelic homologus recombination (NAHR), which in turn, produces novel chromosomal rearrangements and copy number changes. Therefore, CNVRs that overlap with SDs typically display high frequencies as compared to the CNVRs that do not overlap SDs. Accordingly, we found an enrichment of highly frequent CNVRs in cattle SDs as previously observed in human, mice, and apes (Redon et al., 2006; Gazave et al., 2011; Pezer et al., 2015).

Recently, Sudmant et al. (2015), while analyzing CNV patterns across different human populations and a Denisovan sample, identified large duplications that introgressed from the extinct Denisovan lineage exclusively into Oceanic population, and also were present at high frequencies. Since north-western European cattle breeds harbor high frequency of aurochs’ specific alleles, probably as a result of secondary aurochs introgression (Park et al., 2015; Upadhyay et al., 2016), we investigated whether animals from these regions carry any unique CNVs. However, V_st_ based analysis did not identify any region specific CNV that might have introgressed from aurochs during secondary contact, i.e., CNV present only in animals of certain regions as a result of aurochs introgression. In the future, the availability of high-coverage sequence from archaic aurochs samples might aid researchers in identification of ancient CNVs in the genome of European cattle. Additionally, V_st_ based analysis also did not identify any breed-group differential CNVs, when contrasting HF (commercial breed) against IBR or BAI animals. This observation is consistent with a recent study on bovine population structure, where authors reported only few lineage-specific CNVs in breeds from the same continent, i.e., Holstein and Angus cattle breeds (Xu et al., 2016). However, quite a few breed-group differentiated CNVs between HF and BRI were identified, except a few, all of which turned out to be false positive CNVs.

### Copy Number Variable Genes

Cattle genomes display enrichment of CNVs in genes related to immune response and environmental interaction such as sensory perceptions of smell and chemical stimuli. Many of these immune related genes appeared to be copy number variable between different cattle breeds. These variations may explain inter-population differences in immunological response to different clinical conditions. For example, *BoLA-DRB3* locus which partially lies within a high frequency complex CNVR (id: CNVr1586, **Supplementary Table S8**) has been associated with differential response to various clinical conditions such as Mastitis and Bovine leukemia virus infection in various cattle breeds (Rupp et al., 2007; Yoshida et al., 2012). Another interesting example is the *CIITA* gene, which lies within a high frequency gain CNVR identified in multiple breeds (id: CNVr1680, **Supplementary Table S8**) and which was found to be duplicated in Angus cattle that showed nematode resistance (Liu et al., 2011). On the other hand, enrichment of CNVs in genes related to sensory perception of smell is either indicative of physiological requirements of domestic animals as described previously in case of pig (Paudel et al., 2013) or can, alternatively, be the result of drift due to random duplication and deletion of olfactory genes (OR) (Nozawa and Nei, 2007). Similar overrepresentation of CNVs in immune related genes and OR genes was reported previously in various species of domestic animals (Paudel et al., 2013; Liu et al., 2013; da Silva et al., 2016). The most frequent CNVR displayed variable copy numbers and covered genes similar to *ABCC4* in human. The *ABCC4* genes encode a protein related to ATP-binding cassette (ABC) transporters which transport various molecules across extra- and intra-cellular membranes. Previous studies have reported association between *ABCC4* genes and phenotypic traits related to disease resistance and feed efficiency in cattle (Liu et al., 2011; Chen et al., 2012). Interestingly, Lee et al. (2013), using whole genome sequencing data of Hanwoo cattle, reported a very high number of non-synonymous SNPs, splice-site variants, and coding indels in *ABCC4* gene. These observations imply that either *ABCC4* gene has evolved into multiple copies for environmental adaptation, or, alternatively, mis-assembly at chromosome 12 led to distortion of signal intensity ratio resulting in detection of false CNVRs. The CNVR1206 that covered the *MTHFSD* gene, has been associated with milk protein yield in Spanish HF cattle (Ben Sassi et al., 2016), while the *GTF2I* gene that lies within CNVR1703, has been identified as a candidate gene related to traits associated with feed conversion efficiency in chicken (Reyer et al., 2015). Novel CNVRs, i.e., CNVRs that were identified only in the present study, spanned important genes such as *MSH4* and *ATF2* etc. The *MSH4* gene encodes a protein essential for reciprocal recombination and proper segregation of homologous chromosomes at meiosis. Additionally, deficiency of *MSH4* gene, which is covered by CNVr1975 (**Supplementary Table S8**) and identified only in two animals, has been associated with impaired gamete formations in laboratory mice (Kneitz et al., 2000). Recently, Ma et al. (2015) and Kadri et al. (2016) also have associated the *MSH4* gene with recombination rate in cattle. Also, deficiency of the *ATF2* gene, which is partially covered by CNVr1217 and identified only in four animals, led to early postnatal lethality in laboratory mice (Bhoumik and Ronai, 2008). Since both these CNVRs (id: CNVr1975 and CNVr1217) have been identified in low frequency and in heterozygous deletion state, the hypothesis of purifying selection against such deleterious CNVs cannot be ruled out.

Recently, Durkin et al. (2012) have shown that a duplication of a *KIT* gene segment from chromosome 6 and its aberrant insertion on chromosome 29 led to the “color-sided” white coat color phenotype in Belgian blue cattle. Additionally, Brenig et al. (2013) identified the Belgian-blue type allele (Cs29) in White Park and Galloway cattle. Interestingly, they also suggested a dose dependent effect of Cs29 in which heterozygous (Cs29/wild allele) animals exhibited variable degrees of pigmented spots on white body trunk and homozygous (Cs29/Cs29) animals produced no pigmentation on the body trunk. In our study, we show the presence of the Belgian Blue type allele in EL cattle, most likely introduced in the EL due to cross breeding with other cattle breeds such as Galloway and White park that also carry the same allele. In addition, as both EL animals were homozygous for Cs29 and as this breed harbors low diversity, we hypothesize that frequency of Cs29 allele is high in this breed.

In summary, we utilized signal intensity data from Bovine Illumina HD genotyping array to identify CNV in cattle populations sampled from different regions of Europe. The comparative evaluation indicated a higher abundance of CNV counts in British and Balkan-Italian cattle breeds, probably because of high historical effective population size or relaxation on purifying selection of slightly deleterious CNVs. Also, clustering based on CNV regions displayed low population differentiation indicating the effect of transient CNVs or CNV hot-spot region. Functional analysis revealed enrichment of CNVR in genes related to immunological responses and environmental interaction such as sensory perceptions of smell and chemical stimuli. In addition, we also detected a CNV overlapping the *Kit* gene in EL cattle which has been identified previously in Belgian blue, white park and Galloway cattle and associated with color-sidedness.

## Data Accessibility

The total signal intensity (LRR) and B Allele frequency (BAF) data of majority of the samples used in the present study are available from the Dryad Digital Repository: 10.5061/dryad.s57d6. The LRR and BAF data of HF samples used in the study are available to interested researcher upon the request.

## Author Contributions

MU, RC, and MG conceived the study, its design and coordination. MV, PA-M, VB, SD, JG, CG, and JK identified pure bred animals and carried out sampling. MU and VdS identified CNV and performed filtration for subsequent analysis. MU performed all the downstream analysis. MU drafted the manuscript, MG, RC, H-JM, PA-M, VB, SD, JG, CG, VdS, and JK provided critical remarks on the content and all authors read and approved the manuscript.

## Conflict of Interest Statement

The authors declare that the research was conducted in the absence of any commercial or financial relationships that could be construed as a potential conflict of interest.

## Footnotes

1 http://frodo.wi.mit.edu/primer3/

2 http://genomes.urv.cat/UPGMA/

3 http://hgdownload.soe.ucsc.edu/goldenPath/bosTau8/bigZips/

## Supplementary Material

TABLE S1 Sampling information: the first column is breed code, the second column is breed name, the third and fourth columns displays information about country and region of origin for the breed, respectively, and the fifth column displays number of samples collected per breed. ALP, Alpine; BRI, British and Irish; NLD, Dutch; JE, Jersey; IBR, Iberian; BAI,Balkan and Italy.

TABLE S2 Detail information about chemical reagents, primer, and results of qPCR used for CNV validation.

TABLE S3 Distribution of CNVs into different classes for each animal.

TABLE S4 List of all CNVs including sample id and other remarks.

TABLE S5 Detail information about all Copy number variable regions (CNVR) including its frequency and sample information.

TABLE S6 Information about segmental duplication found to be overlapped with CNVR identified in the present study.

TABLE S7 List of false positive CNVR identified as breed group differentiated based on V_st_ approach.

TABLE S8 Information about Ensembl genes found to be overlapped with CNVR identified in the present study.

TABLE S9 Information about Ensembl genes found to be overlapped with unique CNVR that are only identified in the present study.

TABLE S10 Detail about gene ontology category significantly overrepresented in CNVR.

TABLE S11 List of all unique CNVRs which are identified only in the present study (after comparing with the previous studies).

## References

[B1] Bae J. S. Cheong H. S. Kim L. H. NamGung S. Park T. J. Chun J.-Y. (2010). Identification of copy number variations and common deletion polymorphisms in cattle. *BMC Genomics* 11:232 10.1186/1471-2164-11-232 PMC2859865 20377913

[B2] Ben Sassi N. González-Recio Ó. de Paz-del Río R. Rodríguez-Ramilo S. T. Fernández A. I. (2016). Associated effects of copy number variants on economically important traits in Spanish Holstein dairy cattle. *J. Dairy Sci.* 99 6371–6380. 10.3168/jds.2015-10487 27209136

[B3] Bhoumik A. Ronai Z. (2008). ATF2: a transcription factor that elicits oncogenic or tumor suppressor activities. *Cell Cycle* 7 2341–2345. 10.4161/cc.6388 18677098

[B4] Bickhart D. M. Hou Y. Schroeder S. G. Alkan C. Cardone M. F. Matukumalli L. K. (2012). Copy number variation of individual cattle genomes using next-generation sequencing. *Genome Res.* 22 778–790. 10.1101/gr.133967.111 22300768 PMC3317159

[B5] Bickhart D. M. Xu L. Hutchison J. L. Cole J. B. Null D. J. Schroeder S. G. (2016). Diversity and population-genetic properties of copy number variations and multicopy genes in cattle. *DNA Res.* 23 253–262. 10.1093/dnares/dsw013 27085184 PMC4909312

[B6] Boussaha M. Esquerré D. Barbieri J. Djari A. Pinton A. Letaief R. (2015). Genome-wide study of structural variants in bovine Holstein, Montbeliarde and Normande dairy breeds. *PLoS ONE* 10:e0135931 10.1371/journal.pone.0135931 PMC4552564 26317361

[B7] Brenig B. Beck J. Floren C. Bornemann-Kolatzki K. Wiedemann I. Hennecke S. (2013). Molecular genetics of coat colour variations in White Galloway and White Park cattle. *Anim. Genet.* 44 450–453. 10.1111/age.12029 23418861

[B8] Chen C. Qiao R. Wei R. Guo Y. Ai H. Ma J. (2012). A comprehensive survey of copy number variation in 18 diverse pig populations and identification of candidate copy number variable genes associated with complex traits. *BMC Genomics* 13:733 10.1186/1471-2164-13-733 PMC3543711 23270433

[B9] da Silva V. H. De Almeida Regitano L. C. Geistlinger L. Pertille F. Giachetto P. F. Brassaloti R. A. (2016). Genome-wide detection of CNVs and their association with meat tenderness in Nelore cattle. *PLoS ONE* 11:e0157711 10.1371/journal.pone.0157711 PMC4922624 27348523

[B10] Decker J. E. McKay S. D. Rolf M. M. Kim J. W. Molina Alcalá A. Sonstegard T. S. (2014). Worldwide patterns of ancestry, divergence, and admixture in domesticated cattle. *PLoS Genet.* 10:e1004254 10.1371/journal.pgen.1004254 PMC3967955 24675901

[B11] Dumas L. Kim Y. H. Karimpour-Fard A. Cox M. Hopkins J. Pollack J. R. (2007). Gene copy number variation spanning 60 million years of human and primate evolution. *Genome Res.* 17 1266–1277. 10.1101/gr.6557307 17666543 PMC1950895

[B12] Durkin K. Coppieters W. Drögemüller C. Ahariz N. Cambisano N. Druet T. (2012). Serial translocation by means of circular intermediates underlies colour sidedness in cattle. *Nature* 482 81–84. 10.1038/nature10757 22297974

[B13] Fadista J. Thomsen B. Holm L.-E. Bendixen C. (2010). Copy number variation in the bovine genome. *BMC Genomics* 11:284 10.1186/1471-2164-11-284 PMC2902221 20459598

[B14] Gazave E. Darré F. Morcillo-suarez C. Ventura M. Catacchio C. R. Alkan C. (2011). Copy number variation analysis in the great apes reveals species-specific patterns of structural variation. *Genome Res.* 21 1626–1639. 10.1101/gr.117242.110 21824994 PMC3202280

[B15] Gonzalez E. Kulkarni H. Bolivar H. Mangano A. Sanchez R. Catano G. (2005). The influence of CCL3L1 gene-containing segmental duplications on HIV-1/AIDS susceptibility. *Science* 307 1434–1440. 10.1126/science.1101160 15637236

[B16] Hou Y. Bickhart D. M. Hvinden M. L. Li C. Song J. Boichard D. A. (2012). Fine mapping of copy number variations on two cattle genome assemblies using high density SNP array. *BMC Genomics* 13:376 10.1186/1471-2164-13-376 PMC3583728 22866901

[B17] Hou Y. Liu G. Bickhart D. Cardone M. Wang K. Kim E. (2011). Genomic characteristics of cattle copy number variations. *BMC Genomics* 12:127 10.1186/1471-2164-12-127 PMC3053260 21345189

[B18] Jiang L. Jiang J. Wang J. Ding X. Liu J. Zhang Q. (2012). Genome-wide identification of copy number variations in Chinese Holstein. *PLoS ONE* 7:e48732 10.1371/journal.pone.0048732 PMC3492429 23144949

[B19] Jiang L. Jiang J. Yang J. Liu X. Wang J. Wang H. (2013). Genome-wide detection of copy number variations using high-density SNP genotyping platforms in Holsteins. *BMC Genomics* 14:131 10.1186/1471-2164-14-131 PMC3639935 23442346

[B20] Kadri N. K. Harland C. Faux P. Cambisano N. Karim L. Coppieters W. (2016). Coding and noncoding variants in HFM1, MLH3, MSH4, MSH5, RNF212, and RNF212B affect recombination rate in cattle. *Genome Res.* 26 1323–1332. 10.1101/gr.204214.116 27516620 PMC5052053

[B21] Khaja R. MacDonald J. R. Zhang J. Scherer S. W. (2006). Methods for identifying and mapping recent segmental and gene duplications in eukaryotic genomes. *Methods Mol. Biol.* 338 9–20. 10.1385/1-59745-097-9:9 16888347

[B22] Kijas J. W. Barendse W. Barris W. Harrison B. McCulloch R. McWilliam S. (2011). Analysis of copy number variants in the cattle genome. *Gene* 482 73–77. 10.1016/j.gene.2011.04.011 21620936

[B23] Kneitz B. Cohen P. E. Avdievich E. Zhu L. Kane M. F. Hou H. (2000). MutS homolog 4 localization to meiotic chromosomes is required for chromosome pairing during meiosis in male and female mice. *Genes Dev.* 14 1085–1097. 10.1101/gad.14.9.1085 10809667 PMC316572

[B24] Lee K.-T. Chung W.-H. Lee S.-Y. Choi J.-W. Kim J. Lim D. (2013). Whole-genome resequencing of Hanwoo (Korean cattle) and insight into regions of homozygosity. *BMC Genomics* 14:519 10.1186/1471-2164-14-519 PMC3750754 23899338

[B25] Liu G. E. Brown T. Hebert D. A. Cardone M. F. Hou Y. Choudhary R. K. (2011). Initial analysis of copy number variations in cattle selected for resistance or susceptibility to intestinal nematodes. *Mamm. Genome* 22 111–121. 10.1007/s00335-010-9308-0 21125402

[B26] Liu G. E. Hou Y. Zhu B. Liu G. E. Hou Y. Zhu B. (2010). Analysis of copy number variations among diverse cattle breeds. *Genome Res.* 20 693–703. 10.1101/gr.105403.110 20212021 PMC2860171

[B27] Liu J. Zhang L. Xu L. Ren H. Lu J. Zhang X. (2013). Analysis of copy number variations in the sheep genome using 50K SNP BeadChip array. *BMC Genomics* 14:229 10.1186/1471-2164-14-229 PMC3626776 23565757

[B28] Lupski J. R. (2007a). An evolution revolution provides further revelation. *Bioessays* 29 1182–1184. 10.1002/bies.20686 18008371

[B29] Lupski J. R. (2007b). Genomic rearrangements and sporadic disease. *Nat. Genet.* 39 S43–S47. 10.1038/ng2084 17597781

[B30] Ma L. O’Connell J. R. VanRaden P. M. Shen B. Padhi A. Sun C. (2015). Cattle sex-specific recombination and genetic control from a large pedigree analysis. *PLoS Genet.* 11:e1005387 10.1371/journal.pgen.1005387 PMC4634960 26540184

[B31] Mi H. Lazareva-Ulitsky B. Loo R. Kejariwal A. Vandergriff J. Rabkin S. (2005). The PANTHER database of protein families, subfamilies, functions and pathways. *Nucleic Acids Res.* 33 284–288. 10.1093/nar/gki078 PMC540032 15608197

[B32] Norris B. J. Whan V. A. (2008). A gene duplication affecting expression of the ovine ASIP gene is responsible for white and black sheep. *Genome Res.* 18 1282–1293. 10.1101/gr.072090.107 18493018 PMC2493430

[B33] Nozawa M. Nei M. (2007). Evolutionary dynamics of olfactory receptor genes in *Drosophila* species. *Proc. Natl. Acad. Sci. U.S.A.* 104 7122–7127. 10.1073/pnas.0702133104 17438280 PMC1855360

[B34] Park S. D. E. Magee D. A. McGettigan P. A. Teasdale M. D. Edwards C. J. Lohan A. J. (2015). Genome sequencing of the extinct Eurasian wild aurochs, *Bos primigenius*, illuminates the phylogeography and evolution of cattle. *Genome Biol.* 16:234 10.1186/s13059-015-0790-2 PMC4620651 26498365

[B35] Paudel Y. Madsen O. Megens H.-J. Frantz L. A. F. Bosse M. Bastiaansen J. W. M. (2013). Evolutionary dynamics of copy number variation in pig genomes in the context of adaptation and domestication. *BMC Genomics* 14:449 10.1186/1471-2164-14-449 PMC3716681 23829399

[B36] Pezer Ž. Harr B. Teschke M. Babiker H. Tautz D. (2015). Divergence patterns of genic copy number variation in natural populations of the house mouse (*Mus musculus domesticus*) reveal three conserved genes with major population-specific expansions. *Genome Res.* 25 1114–1124. 10.1101/gr.187187.114 26149421 PMC4509996

[B37] Pielberg G. Olsson C. Syvänen A. C. Andersson L. (2002). Unexpectedly high allelic diversity at the KIT locus causing dominant white color in the domestic pig. *Genetics* 160 305–311. 11805065 10.1093/genetics/160.1.305 PMC1461930

[B38] Pinto D. Darvishi K. Shi X. Rajan D. Rigler D. Fitzgerald T. (2011). Comprehensive assessment of array-based platforms and calling algorithms for detection of copy number variants. *Nat. Biotechnol.* 29 512–520. 10.1038/nbt.1852 21552272 PMC3270583

[B39] Redon R. Ishikawa S. Fitch K. R. Feuk L. Perry G. H. Andrews T. D. (2006). Global variation in copy number in the human genome. *Nature* 444 444–454. 10.1038/nature05329 17122850 PMC2669898

[B40] Reyer H. Hawken R. Murani E. Ponsuksili S. Wimmers K. (2015). The genetics of feed conversion efficiency traits in a commercial broiler line. *Sci. Rep.* 5:16387 10.1038/srep16387 PMC4639841 26552583

[B41] Rupp R. Hernandez A. Mallard B. A. (2007). Association of bovine leukocyte antigen (BoLA) DRB3.2 with immune response, mastitis, and production and type traits in Canadian Holsteins. *J. Dairy Sci.* 90 1029–1038. 10.3168/jds.S0022-0302(07)71589-8 17235182

[B42] Salmon Hillbertz N. H. C. Isaksson M. Karlsson E. K. Hellmén E. Pielberg G. R. Savolainen P. (2007). Duplication of *FGF3, FGF4, FGF19* and *ORAOV1* causes hair ridge and predisposition to dermoid sinus in Ridgeback dogs. *Nat. Genet.* 39 1318–1320. 10.1038/ng.2007.4 17906623

[B43] Sasaki S. Watanabe T. Nishimura S. Sugimoto Y. (2016). Genome-wide identification of copy number variation using high-density single-nucleotide polymorphism array in Japanese Black cattle. *BMC Genet.* 17:26 10.1186/s12863-016-0335-z PMC4727303 26809925

[B44] Sharp A. J. Locke D. P. Mcgrath S. D. Cheng Z. Bailey J. A. Vallente R. U. (2005). Segmental duplications and copy-number variation in the human genome. *Am. J. Hum. Genet.* 77 78–88. 10.1086/431652 15918152 PMC1226196

[B45] Stankiewicz P. Lupski J. R. (2002). Genome architecture, rearrangements and genomic disorders. *Trends Genet.* 18 74–82. 10.1016/S0168-9525(02)02592-1 11818139

[B46] Stankiewicz P. Lupski J. R. (2010). Structural variation in the human genome and its role in disease. *Annu. Rev. Med.* 61 437–455. 10.1146/annurev-med-100708-204735 20059347

[B47] Stothard P. Choi J.-W. Basu U. Sumner-Thomson J. M. Meng Y. Liao X. (2011). Whole genome resequencing of black Angus and Holstein cattle for SNP and CNV discovery. *BMC Genomics* 12:559 10.1186/1471-2164-12-559 PMC3229636 22085807

[B48] Sudmant P. H. Mallick S. Nelson B. J. Krumm N. Huddleston J. Coe B. P. (2015). Global diversity, population stratification, and selection of human copy number variation. *Science* 349:aab3761 10.1126/science.aab3761 PMC4568308 26249230

[B49] Upadhyay M. R. Chen W. Lenstra J. A. Goderie C. R. J. MacHugh D. E. Park S. D. E. (2016). Genetic origin, admixture and population history of aurochs (*Bos primigenius*) and primitive European cattle. *Heredity* 118 169–176. 10.1038/hdy.2016.79 27677498 PMC5234481

[B50] Wang M. D. Dzama K. Hefer C. A. Muchadeyi F. C. (2015). Genomic population structure and prevalence of copy number variations in South African Nguni cattle. *BMC Genomics* 16:894 10.1186/s12864-015-2122-z PMC4632335 26531252

[B51] Wang K. Li M. Hadley D. Liu R. Glessner J. Grant S. F. A. (2007). PennCNV: an integrated hidden markov model designed for high-resolution copy number variation detection in whole-genome SNP genotyping data. *Genome Res.* 17 1665–1674. 10.1101/gr.6861907 17921354 PMC2045149

[B52] Xu L. Cole J. B. Bickhart D. M. Hou Y. Song J. VanRaden P. M. (2014). Genome wide CNV analysis reveals additional variants associated with milk production traits in Holsteins. *BMC Genomics* 15:683 10.1186/1471-2164-15-683 PMC4152564 25128478

[B53] Xu L. Hou Y. Bickhart D. M. Zhou Y. Hay E. H. A. Song J. (2016). Population-genetic properties of differentiated copy number variations in cattle. *Sci. Rep.* 6:23161 10.1038/srep23161 PMC4804293 27005566

[B54] Yoshida T. Furuta H. Kondo Y. Mukoyama H. (2012). Association of BoLA-DRB3 alleles with mastitis resistance and susceptibility in Japanese Holstein cows. *Anim. Sci. J.* 83 359–366. 10.1111/j.1740-0929.2011.00972.x 22574787

[B55] Zarrei M. MacDonald J. R. Merico D. Scherer S. W. (2015). A copy number variation map of the human genome. *Nat. Rev. Genet.* 16 172–183. 10.1038/nrg3871 25645873

[B56] Zhang F. Gu W. Hurles M. E. Lupski J. R. (2009). Copy number variation in human health, disease, and evolution. *Annu. Rev. Genomics Hum. Genet.* 10 451–481. 10.1146/annurev.genom.9.081307.164217 19715442 PMC4472309

[B57] Zhang L. Jia S. Plath M. Huang Y. Li C. Lei C. (2015). Impact of parental *Bos taurus* and *Bos indicus* origins on copy number variation in traditional Chinese cattle breeds. *Genome Biol. Evol.* 7 2352–2361. 10.1093/gbe/evv151 26260653 PMC4558867

[B58] Zhang L. Jia S. Yang M. Xu Y. Li C. Sun J. (2014). Detection of copy number variations and their effects in Chinese bulls. *BMC Genomics* 15:480 10.1186/1471-2164-15-480 PMC4073501 24935859

[B59] Zhang Q. Ma Y. Wang X. Zhang Y. Zhao X. (2014). Identification of copy number variations in Qinchuan cattle using BovineHD Genotyping Beadchip array. *Mol. Genet. Genomics* 290 319–327. 10.1007/s00438-014-0923-4 25248638

[B60] Zhou Y. Utsunomiya Y. T. Xu L. Hay el E. H. Bickhart D. M. Sonstegard T. S. (2016). Comparative analyses across cattle genders and breeds reveal the pitfalls caused by false positive and lineage-differential copy number variations. *Sci. Rep.* 6:29219 10.1038/srep29219 PMC4933914 27381368

[B61] Zhu C. Fan H. Yuan Z. Hu S. Ma X. Xuan J. (2016). Genome-wide detection of CNVs in Chinese indigenous sheep with different types of tails using ovine high-density 600K SNP arrays. *Sci. Rep.* 6:27822 10.1038/srep27822 PMC4901276 27282145

