# Supplementary material for: Distribution and Functionality of Copy Number Variation across European Cattle Populations
Source: Front Genet. 2017 Aug 23;8:108. doi: 10.3389/fgene.2017.00108 (PMC5572341; doi:10.3389/fgene.2017.00108)
Supplement: Supplementary file 2 [file Image_1.PDF]

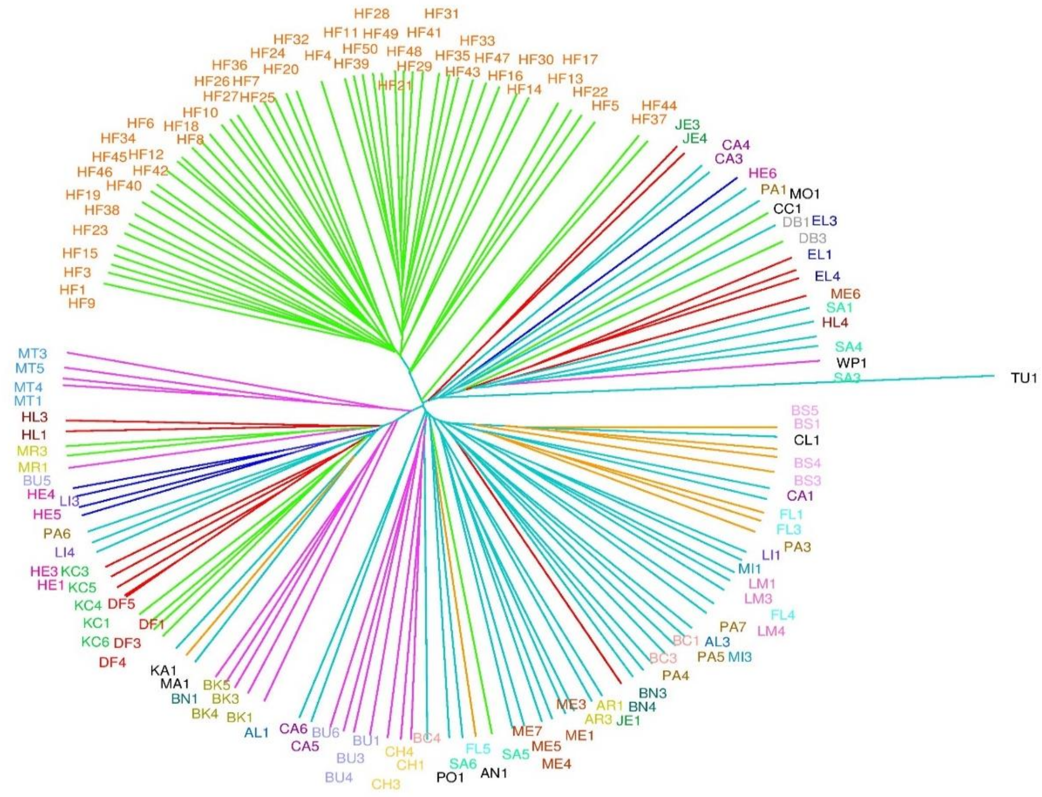

Figure S1. Hierarchical clustering of 149 samples based on all 923 CNVRs. The different colours of sample id represent different breed, while different colours of branch represent different breed group. Green color represents NLD samples, Red color represents BRI samples, Pink color represents BAI samples, Dark yellow color represents ALP samples and Blue color represents IBR samples.

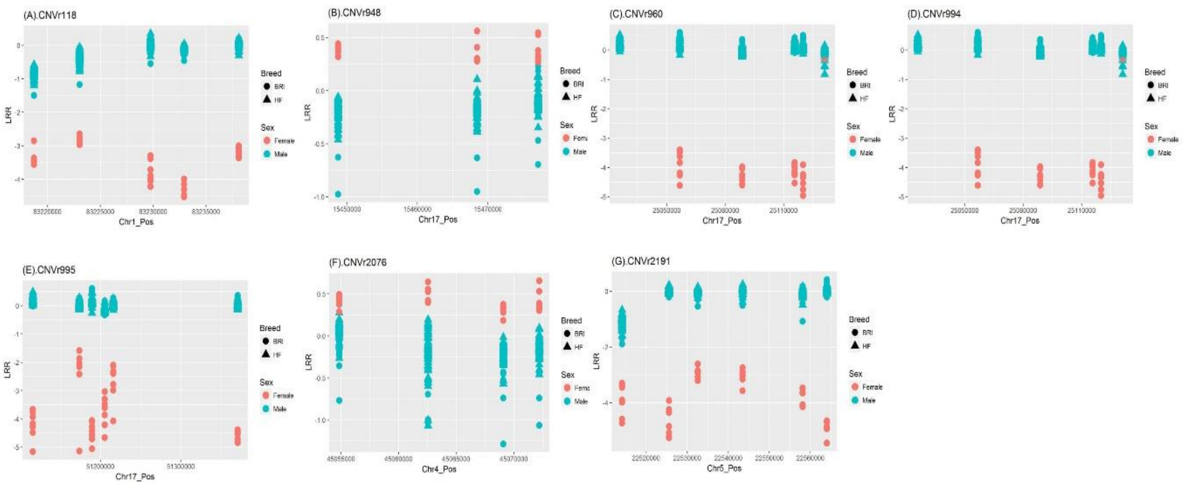

Figure S2. Distribution of LRR values of different SNP probes identified in False positive CNVRs in previous study (Please refer to Zhou et al., (2017) For more details). Some of these CNVRs display high Vst (>0.35) between HF and BRI samples.

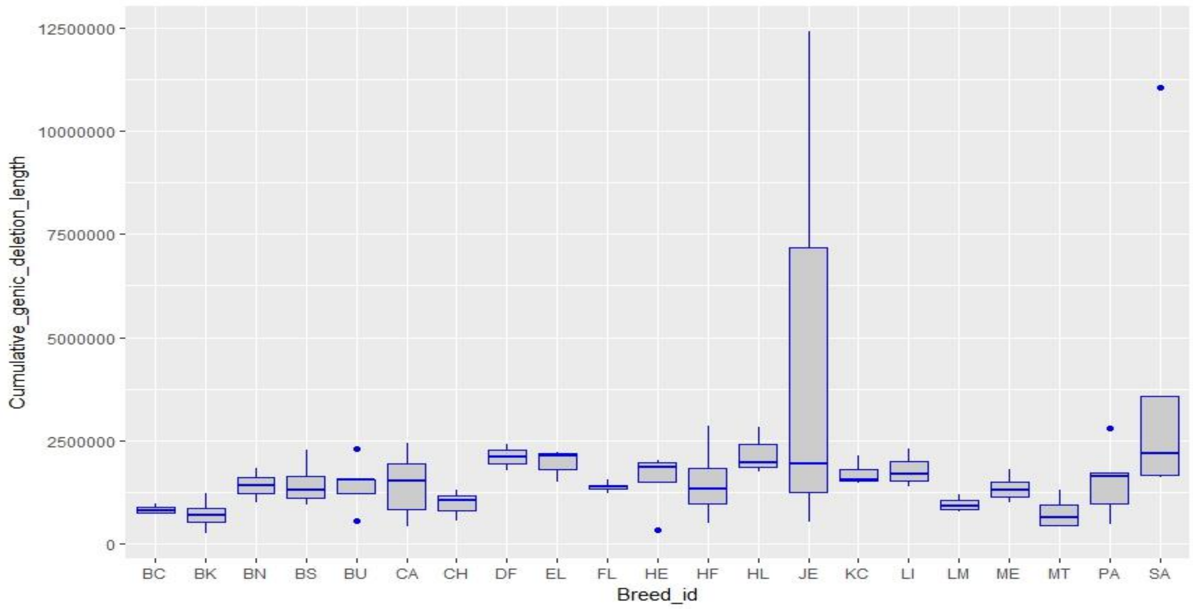

(A).

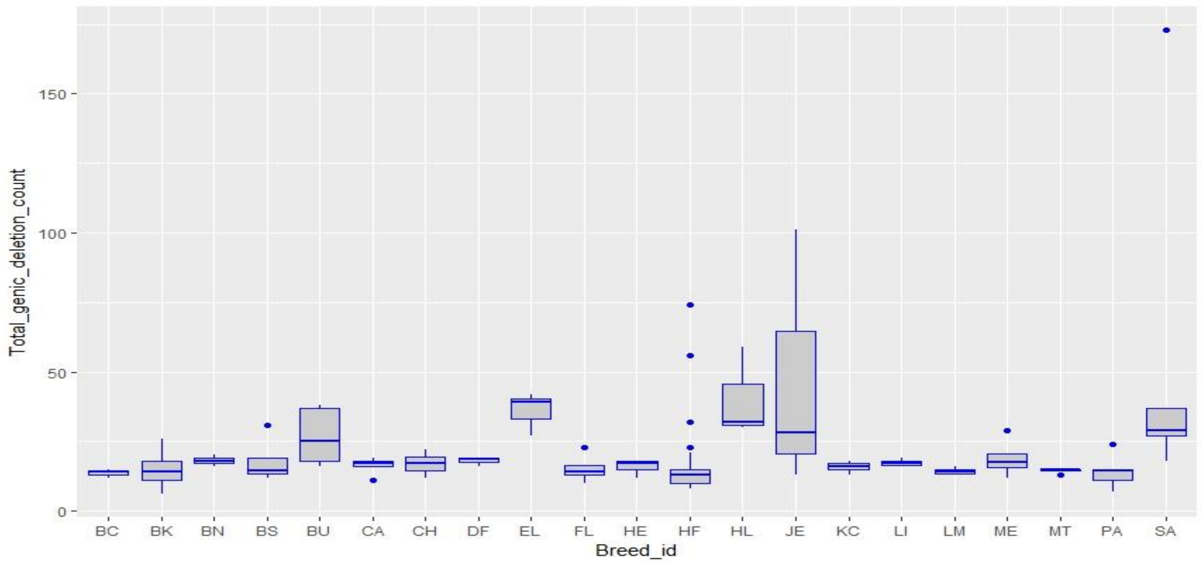

(B).

Figure S3. (A). Representation of genic deletion CNV counts per sample categorized based on it's breed. (B). Representation of length of genome under cumulative genic deletion per sample categorized based on it's breed.
